# Editorial: Targeting key cellular signaling network for cancer chemotherapy and immunotherapy

**DOI:** 10.3389/fimmu.2024.1423917

**Published:** 2024-05-13

**Authors:** Wanshun Li, Xinyue Wang, Dongling Wan, Jiayu Li, Zhendong Jin

**Affiliations:** ^1^ Department of Gastroenterology, Changhai Hospital, Naval Medical University, Shanghai, China; ^2^ College of Basic Medical Science, Naval Medical University, Shanghai, China

**Keywords:** cellular signaling network, chemotherapy, Immunotherapy, Cancer, targeted therapy

In the dynamic field of oncology, the strategic targeting of cellular signaling networks marks a transformative approach in cancer treatment, specifically through chemotherapy and immunotherapy. This focus is based on the premise that understanding and manipulating the intricate signaling pathways that regulate cell growth, proliferation, and death can lead to more effective and less toxic treatment modalities (1, 2). This Research Topic focuses on the research progress of key signal networks in tumor chemotherapy and immunotherapy, with the aim of comprehensively and deeply elucidating the functions and regulatory mechanisms of key signal networks in the process of tumor cell proliferation, metastasis and drug resistance, and contributing to the development of new chemotherapy drugs and targeted therapies.

Cellular signaling networks encompass various pathways that regulate essential cellular activities such as growth, apoptosis, and differentiation. In cancer, these pathways often become aberrant, leading to uncontrolled cell division and tumor progression. Key pathways such as the PI3K/AKT/mTOR, RAS/RAF/MEK/ERK, and JAK/STAT pathways are frequently disrupted in cancerous cells (3). By specifically targeting these pathways, treatments can directly hinder the growth of cancer cells and potentially lead to more successful outcomes. Zhang et al. analyzed the complex molecular terrain of thymic epithelial tumors and important key genes and signaling pathways in tumorigenesis in order to highlight the significant influence of tumor microenvironment on tumor behavior and treatment response.

The traditional approach of chemotherapy, which indiscriminately targets rapidly dividing cells, is being supplemented and sometimes replaced by targeted therapies and immunotherapies. Targeted therapies focus on specific components within signaling pathways, thereby minimizing damage to normal cells and reducing side effects. Immunotherapy, on the other hand, leverages the body’s immune system to fight cancer, utilizing methods like immune checkpoint inhibitors and CAR T-cell therapy (4). These methods have transformed treatment paradigms, particularly for cancers that were previously deemed untreatable with conventional approaches. Huang et al. integrated the literature about neoadjuvant chemotherapy of bladder cancer from 1999 to 2022 published on Web of Science Core Collection (WoSCC). A growing trend in annual publications and citations related to bladder cancer NAC was confirmed in 1836 publications between 1999 and 2022, suggesting that the integration of immunochemotherapy is expected to experience substantial growth in future studies. Fan et al. showed that natural killer cells can secrete IFN-γ and TNF-α or participate in Fas/FasL and TRAIL/TRAILR pathways, mediating ovarian cancer cell death and greatly improving the efficacy of cellular immunotherapy for ovarian cancer. Additionally, Song et al. identified significantly elevated levels of TBC1 Domain Family Member 1 in tumor tissue from glioma patients, which ultimately affecting the effectiveness of anti-tumor immunotherapy and leading to treatment resistance. In addition, targeting TBC1D1 combined with immune checkpoint blockade treatment may enhance the efficacy of anti-tumor immunotherapy, may inhibit tumor progression and improve the survival rate of patients.

The integration of chemotherapy and immunotherapy represents a forefront of current research, aiming to harness the benefits of both approaches. This integration seeks to exploit chemotherapy’s ability to reduce tumor masses and alter the tumor microenvironment in a way that enhances the effectiveness of immunotherapeutic agents. The findings of Alejandro Martin Garcia-Sancho et al. suggest that patients with peripheral blood T-cell lymphoma may benefit from autologous stem cell transplantation, especially those who have progressed to advanced stages of the disease may benefit more than others.

The focus on cellular signaling networks in cancer therapy, particularly through chemotherapy and immunotherapy, reflects a significant shift towards more precise and personalized treatment options. With continuous research and technological advancements, this field is rapidly evolving, offering hope for more effective and less toxic treatment strategies. The growing understanding of cellular signaling and immune dynamics in cancer is paving the way for innovative treatments that promise to reshape the future of cancer care, making it more targeted, effective, and sustainable. This convergence of technology, science, and medicine marks a promising horizon in the ongoing battle against cancer.

## Author contributions

WL: Writing – original draft. XW: Writing – original draft. DW: Writing – original draft. JL: Writing – original draft. ZJ: Writing – review & editing.
